# Post retention strength of apical and conventional coating obturation methods using bioceramic sealer: a laboratory investigation

**DOI:** 10.1186/s12903-023-03778-2

**Published:** 2024-01-02

**Authors:** Benjarat Chanapairin, Sirinya Kulvitit, Chankhrit Sathorn

**Affiliations:** 1 Department of Operative Dentistry, Faculty of Dentistry, Chulalongkorn University, Bangkok, 10330 Thailand; 2 Center of Excellence in Genomics and Precision Dentistry, Faculty of Dentistry, Chulalongkorn university, Bangkok, 10330 Thailand; 3 School of Dentistry, La Trobe University, Melbourne, VIC Australia

**Keywords:** Fibre post, Push out bond strength, Scanning electron microscope, Resin tag, Hybrid layer

## Abstract

**Background:**

Once bioceramic sealer (BCS) enters the dentinal tubules, it cannot be reliably removed. BCS-occupied dentinal tubules reduce fibre post retention strength. Coating gutta-percha with BCS only on the apical portion may improve post retention strength due to increased retention strength between the dentin and resin cement interface. The aim of the study was to test this hypothesis.

**Methods:**

Root canals of 27 extracted human mandibular premolars were instrumented and randomly assigned to three obturation methods: conventional coating (CC), non-coating (NC), and apical coating (AC). The root canals were obturated with gutta-percha to 4 mm from the working length under an operating microscope. After the BCS was completely set, post spaces were prepared, and quartz fibre posts were cemented. The apical 4.5 mm of the roots were removed. Two samples were prepared at the apical, middle, and coronal root levels (one for scanning electron microscope (SEM) study and another for the push out bond strength (PBS) test). After the PBS test, the samples were examined with a stereo microscope to determine the failure mode: dentine-cement (DC), post-cement (PC) and mixed. The PBS data were analysed by One way ANOVA for the specific obturation method effects. Repeated ANOVA was used for the specific effects of the root levels on PBS in different obturation methods.

**Results:**

At all three root levels, more continuous hybrid layers and denser resin tags were found in the NC and AC than the CC group. The AC and NC groups’ PBS was significantly higher than the CC group at the apical 1/3 (*p* = 0.002 and *p* = 0.001) and coronal 1/3 (*p* = 0.016 and *p* = 0.041). The PBS in the CC group at the middle 1/3 was significantly higher than the apical 1/3 (*p* = 0.022). DC failure mode was most commonly found in the CC group, while PC failure mode was found most frequently in the NC and AC groups.

**Conclusions:**

The apical coating obturation method significantly increased PBS over the conventional coating method, potentially reducing fiber post dislodgement. However, this study was only preliminary. Clinical studies are required to confirm the results.

## Introduction

There has been a shift in obturation philosophy from using a gutta-percha to a sealer-based technique with an aid of a bioceramic sealer (BCS) because it is less technique sensitive and less time consuming [1]. Moreover, the root filling quality using either technique was comparable [2].

BCS sealability and bond strength are better than epoxy resin–based sealer (AH Plus; Dentsply Maillefer, Tulsa, OK, USA) [3–5]. This is because BCS can penetrate deep into the dentinal tubules (DTs) owing to its small particle size (< 2 micron). In addition, BCS slightly expands during setting. Moreover, it infiltrates into the intertubular dentine leading to hydroxyapatite formation, which increases bond strength through a micromechanical mechanism [6, 7].

Although sealer dentinal tubule penetration may be favourable from a sealability standpoint, it could compromise fibre post retention because resin cement cannot penetrate the sealer-occupied DTs, which would reduce the dentine-resin cement bond strength [8, 9]. This, in turn, could result in post dislodgement and ultimately clinical failure. In a ten-year survival study of fibre post-retained restorations of endodontically treated teeth, post dislodgement was found in 31% of the cases [10]. This finding indicates that post dislodgement is a meaningful problem. Furthermore, this study was done before the use of BCS was adopted. The use of BCS, which seals the dentinal tubules, might increase the post dislodgment rate.

Currently, there is no reliable and effective way to remove BCS from the root canals and DTs [11–13]. Enlarging a root canal space with post drills and passive ultrasonic irrigation (PUI) has been used unsuccessfully to open the DTs for resin cement [14, 15].

Post retention strength is significantly higher in root canals without BCS compared with BCS [8, 16, 17]. Therefore, a modified obturation method (i.e., apical coating) that leaves the coronal part of the root canals unsmeared by BCS may improve the post retention strength. The aim of the study was to determine if the post retention strength and failure mode were different in the apical and conventional coating obturation methods. The null hypothesis of this study was that there was no significant difference in post retention strength and failure mode between the conventional and apical coating obturation methods.

## Materials and methods

The sample size was calculated based on a previous study [8] using G*Power 3.1 [18] according to the following formula.

$${n}_{1}=\frac{{\left({Z}_{1-\frac{\alpha }{2}}+{Z}_{1-\beta }\right)}^{2}\left[{\sigma }_{1}^{2}+\frac{{\sigma }_{2}^{2}}{r}\right]}{{\varDelta }^{2}}$$

$$r=\frac{{n}_{2}}{{n}_{1}} , \varDelta ={\mu }_{1}-{\mu }_{2}$$

${n}_{1}$= sample size in group 1

${n}_{2}$= sample size in group 2

${\mu }_{1}$= mean in group1

${\mu }_{2}$= mean in group2

${\sigma }_{1}$= standard deviation in group 1

${\sigma }_{2}$= standard deviation in group 2

$\varDelta$= difference between means

$$\alpha$$= significant level

$\beta$= type II error probability

$r$= a ratio between sample sizes in group 2 and group 1

The significance level was set at 0.05, 80% power. The sample size required was 66. Errors in sampling and processing was estimated to be ~ 20%, therefore, a total sample of 81 from 27 teeth was required. Mandibular premolars with complete root formation, were collected immediately following extraction. The study was approved by the Ethics Committee of the Faculty of Dentistry, Chulalongkorn University, Bangkok, Thailand (HREC-DCU 2020-057). The procedures used in this study adhered to the tenets of the Declaration of Helsinki.

### Tooth selection

Non-carious, non-cracked, non-endodontically treated mandibular premolars with one relatively straight root were selected. Bucco-lingual and mesio-distal periapical radiographs were taken. Only teeth with a canal width ratio less than 2 at 5 mm from the apex in the BL/MD direction were included in the study [3, 19]. The teeth were cleaned and immersed in 0.1% Thymol for disinfection. This solution was replaced every two months. The teeth were tested within six months to minimise bond strength testing disruption due to the storage conditions. The crowns were removed. A 15 mm root length was maintained and the working length was set at 14.5 mm. Root canals with an initial apical file size larger than 35 were excluded.

### Instrumentation

**Fig. 1:**
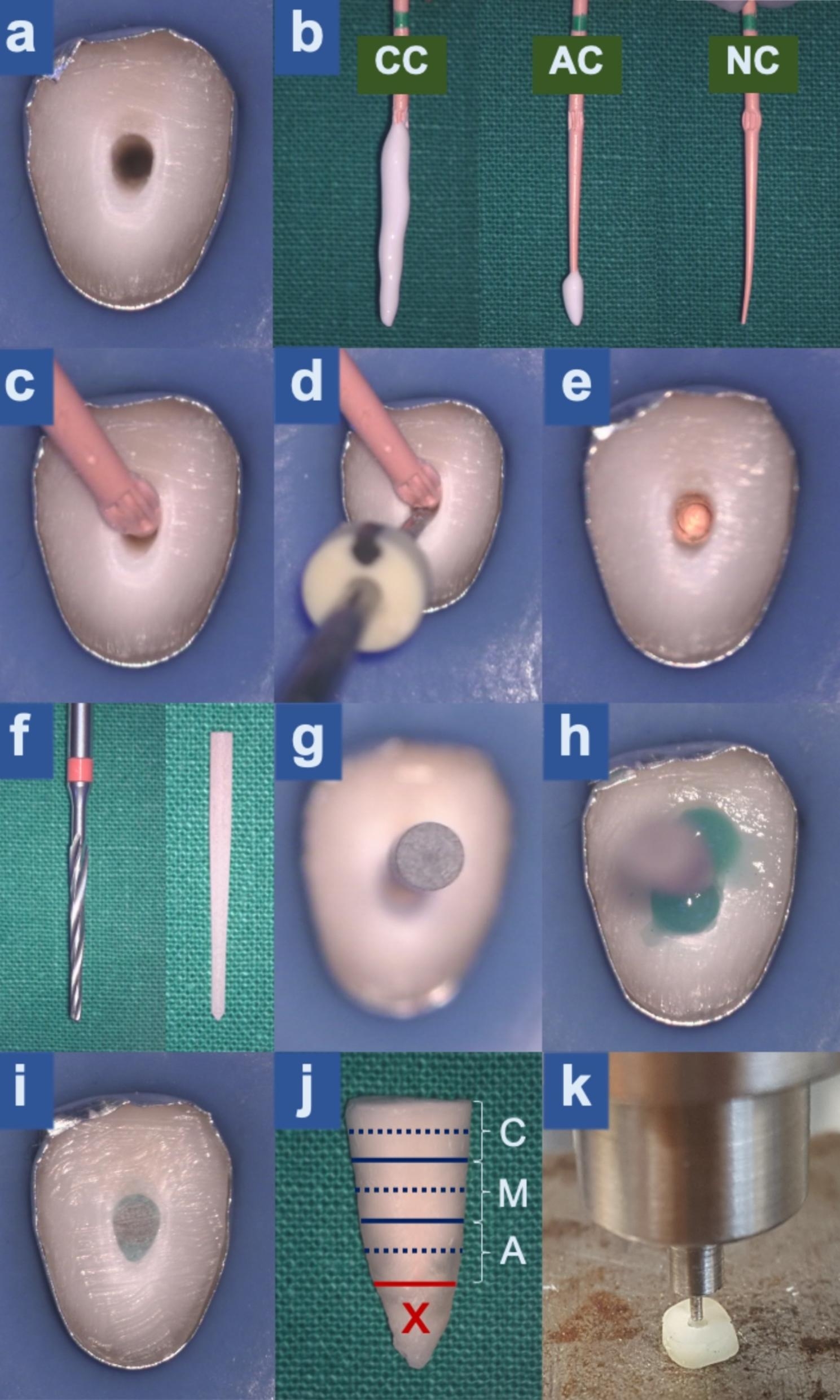
Experimental procedures. **(a)** Root canal after instrumentation. The outer root surface was covered with aluminum foil. **(b)** Matched gutta-percha cones were coated with BCS iRoot SP in CC, AC, and NC groups, respectively. **(c)** A matched gutta-percha cone was inserted into the root canal under an operating microscope. **(d)** A heat carrier was inserted into the canal to 4 mm from the working length using the continuous wave of condensation technique. **(e)** Gutta-percha was condensed with a BL-S Kondenser. The root canal was cleaned with paper points until BCS was not visible under an operating microscope. **(f)** A DT Light-Post Illusion X-RO finishing drill size 1 and a DT Light-Post Illusion X-RO size 1. **(g)** A fibre post was tried-in. **(h)** The post was fixed with Bond 1 as a primer and adhesive and Blue Build-it F.R. dual cure core material following the manufacturer’s recommendations. **(i)** Root canal after post fixation and removal of excess material. **(j)** Sample preparation: The red solid line and red cross indicate the apical 4 mm of the root was removed. The blue solid line indicates the dividing line between each third of the root. ‘C’ indicates the cervical third, ‘M’ indicates the middle third, and ‘A’ indicates the apical third of the root. The blue dashed line indicates the dividing line between specimen preparation for SEM study and PBS test. **(k)** PBS test: A pin was set at the center of the post and loaded in the apico-coronal direction at the rate of 1 mm/min until post dislodgement. Images a-j were captured by the same operating microscope with controlled magnification and settings set at 1.6 magnification (13.6-fold magnification)

A glide path was established with #10 and #15 K files (SybronEndo, Kerr Dental, USA). Root canal preparation was completed with WaveOne Gold Medium 35/0.06 (Dentsply Sirona, Ballaigues, Switzerland). Ten millilitres 2.5% Sodium hypochlorite (NaOCl) were used throughout the preparation. Three millilitres 17% EDTA and 10 ml 2.5% NaOCl were used as a final rinse. PUI was performed three times, for 20 s each, during the last NaOCl rinse. The canals were dried with paper points (Fig. 1a). A medium size (50/0.05) heat carrier (Fast-Pack Heating needle, Eighteeth, Shanghai, China) and BL-S Kondenser (B&L Biotech inc., USA) was fit checked at 10.5 mm length (i.e., 4 mm from the working length).

### Randomization

Randomization was conducted by B.C., who was blinded in the obturation stage. The root surfaces were labelled 1 − 27. B.C. used “random sample” in Excel (Microsoft Office Excel 2010; Microsoft Corporation, Redmond, WA, USA) to randomly assign the teeth to the three obturation method groups, and the assigned numbers were kept in sealed envelopes.

### Obturation

Obturation was performed by S.K., who was not involved in the randomization process.

Group 1 Conventional coating (CC)-positive control (*n* = 9): Medium size (35/0.06) matched gutta-percha WaveOne Gold Conform Fit Gutta-Percha cones (Dentsply Sirona) were coated with BCS iRoot SP (Innovative Bioceramix, Vancouver, Canada) on the entire working length and inserted into the root canals (Fig. 1b c).

Group 2 Non coating (NC)-negative control (*n* = 9): Matched gutta-percha cones were used without sealer and inserted into the root canals (Fig. 1b c).

Group 3 Apical coating (AC)-experimental group (*n* = 9): Matched gutta-percha cones were coated with BCS only at the apical 4 mm and inserted into the root canals. This was done under the operating microscope and great care was taken not to smear BCS on the coronal part of the canals (Fig. 1b c).

The canals were obturated using a continuous wave of condensation with an operating microscope set at a magnification of 1.6 (13.6-fold magnification). A heat carrier was inserted into the canal to 4 mm from the working length (Fig. 1d), gutta-percha was condensed with a BL-S Kondenser. The root canals were cleaned with paper points until the BCS was not visible, using an operating microscope set at a magnification of 2.5 (21.25-fold magnification) (Fig. 1e). Cavit (Four mm thick) (3 M ESPE, St Paul, MN, USA) was placed over the canal orifices. The samples were kept at 37 °C in a humid chamber for 24 h allowing the BCS to completely set.

### Post space preparation and post cementation

The post spaces were prepared by B.C. using a DT Light-Post Illusion X-RO finishing drill size 1 (RTD, Saint Egrève, France) (Fig. 1f). The canals were rinsed with saline solution and dried with paper points. Quartz fibre posts, DT Light-Post Illusion X-RO size 1 (RTD), were inserted to the required length (4 mm from the working length) and radiographs were taken to confirm the length (Fig. 1f g). The canals were rinsed with 17% EDTA and 2.5% NaOCl to remove the smear layer. The canals were then rinsed with a freshly prepared 10%W/V Ascorbic acid solution [20, 21]. Finally, the canals were rinsed with deionized water and dried with paper points.

The posts were fixed with total etch adhesive system using Bond 1 as a primer and adhesive (Pentron Clinical Technologies, Orange, CA, USA). Blue Build-it F.R. dual cure core material (Pentron Clinical Technologies) was used following the manufacturer’s recommendations (Fig. 1h i). The root surfaces were covered with aluminum foil before light curing. This was to simulate the clinical condition when light is inaccessible. The canal orifices were covered with resin composite Filtek Z250 XT (3 M ESPE, St Paul, MN, USA).

The samples were kept in a 37 °C water bath for 24 h following the international standard for tensile bond strength testing (ISO/TS 11,405, 2003).

### Sample preparation

The apical 4.5 mm of the roots was removed. The post-containing portion of the roots were cut perpendicular to the long axis of the roots and polished to prepare 1-mm thick root samples. The samples were prepared at the coronal, middle and apical 1/3 levels. Two samples were prepared at each level (one for the scanning electron microscope (SEM) study and one for the push out bond strength (PBS) test) (Fig. 1j).

### SEM analysis

The samples were sequentially polished with 800-, 1200- and 2500-grit sandpaper. Fine polishing was done using 0.05-micron alumina paste. The samples were cleaned in an ultrasonic water bath for 15 min. They were immersed in a 6 M hydrochloric acid solution for 30 s and washed with water to demineralize them [22]. The samples were placed in 1.25% NaOCl for 60 s and washed with water to deproteinize them. They were kept in a desiccator chamber for 24 h [22, 23]. The samples were placed on alumina stubs with a carbon sheet and gold sputter coated. They were then examined using SEM (Quanta250, FEI, USA) at 500X, 1000X, 1600X and 10000X magnification.

### Push out bond strength (PBS)

The sample images were captured with a Stereo Microscope (SZ 61, OLYMPUS, Japan) from the coronal and apical aspects at 4.5X magnification. The coronal radius (r_1_) and apical radius (r_2_) were measured. The test was done immediately after preparation following ISO/TS 11,405, 2003. A Universal testing machine (EZ-S, SHIMADZU, Japan) was used. The coronal side of the sample was placed on the supporting base. A 0.59-mm diameter pin was set at the centre of the post. The pin was loaded in the apico-coronal direction at the rate of 1 mm/min until post dislodgement (Fig. 1k). The dislodgement forces were recorded in Newtons. The PBS was calculated based on the following equation.

PBS (MPa) = Loading force (N)/Area (mm^2^).

$$Area = \pi \times \left( {{r_1} + {r_2}} \right) \times \sqrt {{{\left( {{r_1} - {r_2}} \right)}^2} + {t^2}}$$

t is sample thickness in mm.

### Failure mode analysis

**Fig. 2:**
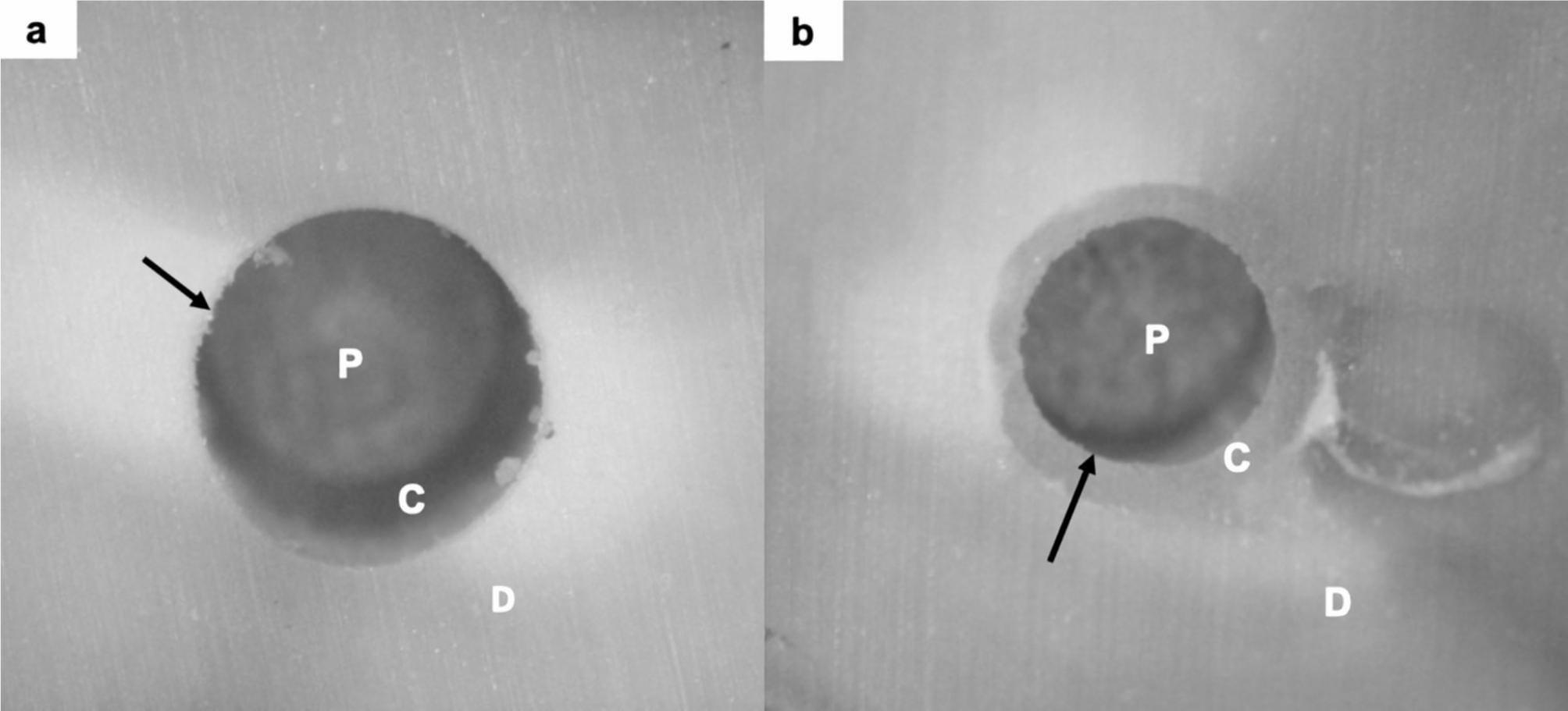
Post-experimental stereo microscope images at 4.5X of DC failure **(a)** and PC failure **(b)**. *D = dentine, C = resin cement layer, P = fibre post*. *Black arrows = failure interface*

After the PBS test, the samples were examined with the Stereo microscope at 4.5X. The failure modes were categorised into three groups. Dentine-Cement (DC) failure was bond failure at the dentine and cement interface. A separation between the dentine and resin cement layer was observed, with the resin cement being firmly attached to the post (Fig. 2a). Post-Cement (PC) failure was bond failure at the post and cement interface. A separation between the post and resin cement layer was observed, with the resin cement being firmly attached to the dentine (Fig. 2b). Mixed failure was a combination of both modes. Cohen’s Kappa coefficient was used to calculate the intra- and inter-observer reliability based on 20 random stereo microscope images of the samples. The readings were done twice one month apart.

During the failure mode analysis, two evaluators (B.C. and S.K.) knew the specimen’s number, but not which obturation method was used. The failure mode analysis was done by two evaluators separately. In case of disagreement, consensus was reached through discussion.

### SEM analysis

Differences in the hybrid layers and resin tags in three obturation methods were examined.

### PBS

SPSS software package (IBM SPSS Statistics 22) was used for statistical analysis. The PBS data distribution was tested using the Shapiro-Wilk test and Levene’s test. Mixed ANOVA was used to test the effects of the obturation methods, root levels and their interactions on PBS. One way ANOVA and Tukey’s post hoc were used for the effects of the obturation methods on PBS at each root level. Repeated ANOVA and Bonferroni pairwise comparison were used for the effects of the root levels on PBS in different obturation methods, with *p* < 0.05 considered significant.

### Failure mode

Descriptive statistics was used to describe the failure modes for the different obturation methods.

## Results

### SEM analysis

**Fig. 3:**
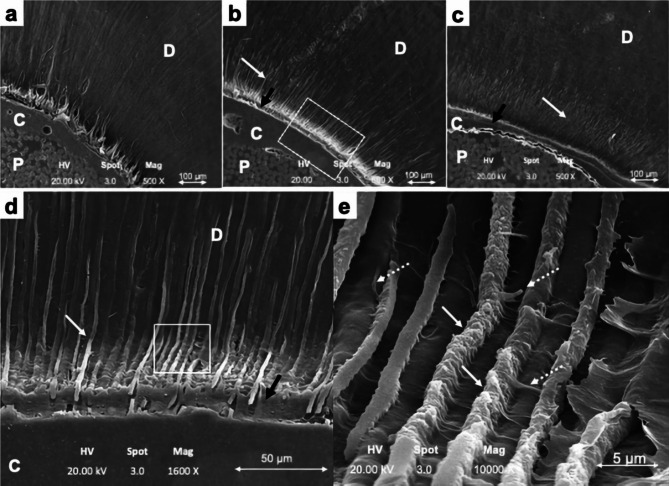
Post-experiment SEM. **(a)** CC group; no hybrid layers nor resin tags under 500x magnification. **(b)** NC group; dense and continuous hybrid layers and resin tags under 500x magnification. **(c)** AC group; dense and continuous hybrid layers and resin tags under 500x magnification. **(d)** NC group; dense and continuous hybrid layers and resin tags under 1600x magnification. **(e)** NC group; resin tags and micro resin tags that branch from main resin tags under 10000x magnification. D = dentine, C = resin cement layer, P = fibre post. *Black arrows = hybrid layer*. *White solid arrows = resin tags*. *White dotted arrows = micro tags*

In the CC group, hybrid layers and resin tags were not found at the apical 1/3 (Fig. 3a). Discontinuous hybrid layers and resin tags were found in the middle 1/3. Continuous hybrid layers and resin tags were present at the coronal 1/3. In the NC group, dense and continuous hybrid layers and resin tags were found in all three levels (Fig. 3b d). At higher magnification (10,000X), micro resin tags were found in the DTs microbranchs (Fig. 3e). Continuous hybrid layers and resin tags were denser in the NC and AC groups (Fig. 3a b c) compared with the CC group at all three root levels.

### PBS

**Fig. 4:**
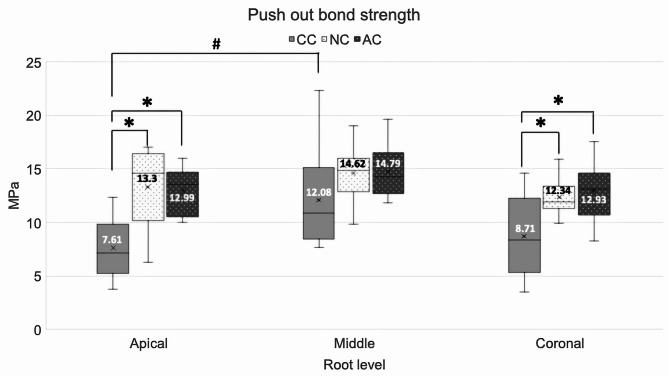
Box and Whisker plot of PBS for the three obturation methods at different root levels, analysed using One-way ANOVA and Tukey’s post hoc test (*p* < 0.05). *Mean PBS, SD at apical of CC (7.61, 2.82), middle of CC (12.08, 4.81), coronal of CC (8.71, 3.94)*. *Mean PBS, SD at apical of NC (13.3, 3.79), middle of NC (14.62, 2.62), coronal of NC (12.34, 1.76)*. *Mean PBS, SD at apical of AC (12.99, 2.2), middle of AC (14.79, 2.51), coronal of AC (12.93, 2.82)*. * and # indicate statistically significant differences (*p* < 0.05)

No samples were lost during the experiment. The PBS data were normally distributed with homogeneity of variance. The obturation methods and root level affected the PBS. There were no interactions between the obturation methods and root levels on the PBS. The PBS in the CC group at the middle 1/3 was significantly higher than the apical 1/3 (mean difference (MD) = 4.48; *p* = 0.022; 95% confidence interval (CI): 0.69 − 8.26). The PBS in the AC and NC groups was not significantly different in all three levels. The NC group’s PBS was significantly higher than the CC group at the apical 1/3 (MD = 5.69; *p* = 0.001; 95% CI: 2.14 − 9.23) and coronal 1/3 (MD = 3.63; *p* = 0.041; 95% CI: 0.13 − 7.14). The PBS in the AC group was significantly higher than the CC group at the apical 1/3 (MD = 5.38; *p* = 0.002; 95% CI: 1.84 − 8.92) and coronal 1/3 (MD = 4.22; *p* = 0.016; 95% CI: 0.72 − 7.73) (Table 1; Fig. 4).

**Table 1:** The mean values of the PBS (MPa) in each group

FRC Method	Root canal level (Mean ± SD)^X^		
	Apical	Middle	Coronal
**Group 1 (CC)** ^**Y**^	7.61 ± 2.82 ^aA^	12.08 ± 4.81 ^bC^	8.71 ± 3.94 ^abD^
**Group 2 (NC)** ^**Y**^	13.30 ± 3.79 ^dB^	14.62 ± 2.62 ^dC^	12.34 ± 1.76 ^dE^
**Group 3 (AC)** ^**Y**^	12.99 ± 2.20 ^cB^	14.79 ± 2.51 ^cC^	12.93 ± 2.82 ^cE^

^X^One way ANOVA and Tukey’s post hoc were used for the effects of obturation methods on PBS at each root level (*p* < 0.05). Different superscript uppercase letters indicate a significant difference between obturation methods in each root canal level

^Y^Repeated ANOVA and Bonferroni pairwise comparison were used for the effects of root levels on PBS in each obturation method (*p* < 0.05). Different superscript lowercase letters indicate significant difference between root levels in each obturation method

### Failure modes

The Kappa score of the interobserver reliability was 1 and intraobserver reliability was 0.93. The reliability was excellent [24].

**Fig. 5:**
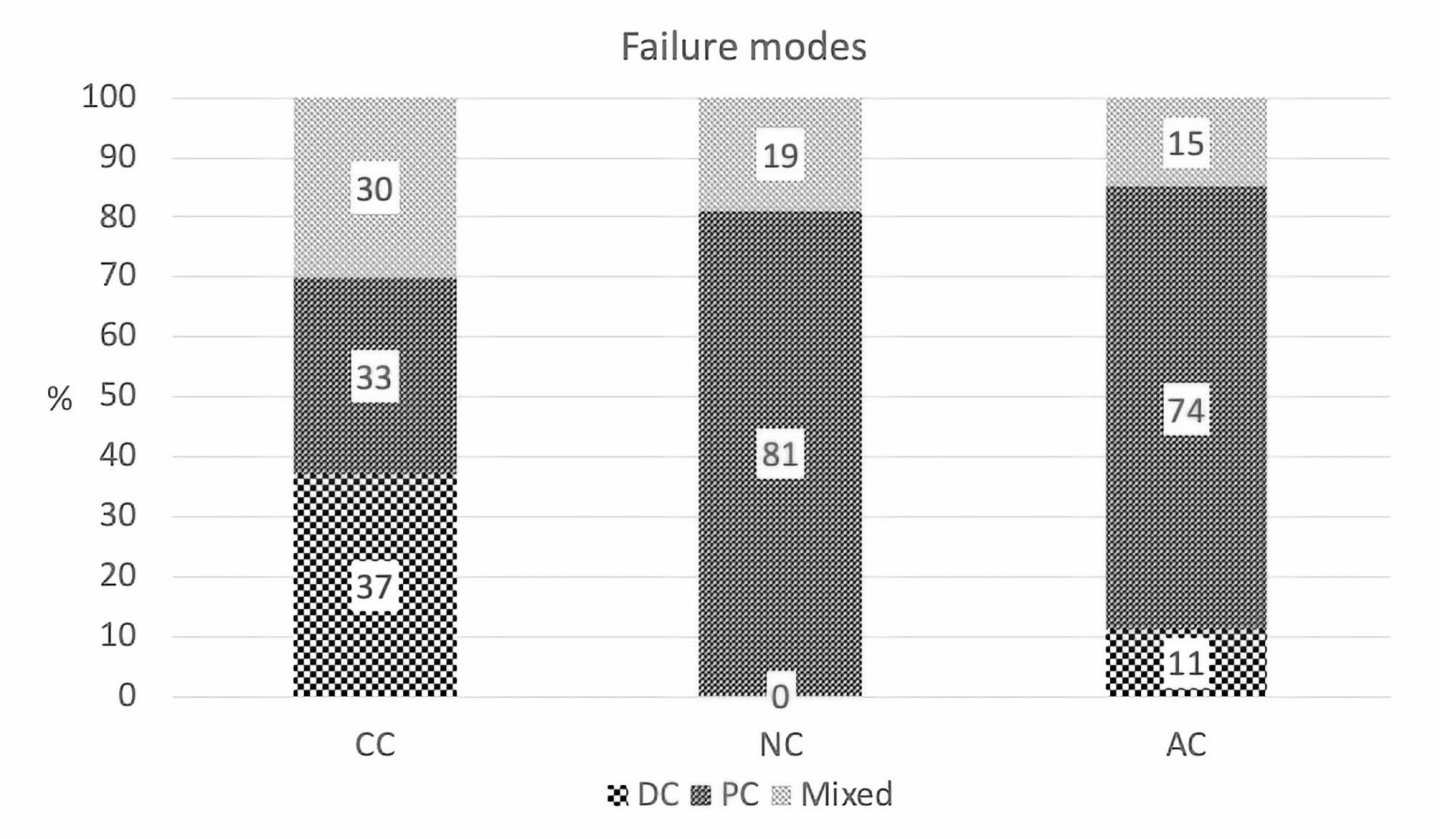
Percent failure modes of three obturation methods

DC failure mode was found most frequently in the CC group followed by PC and mixed mode. PC failure mode was found more often than mixed mode in the NC group. PC failure mode was found most commonly in AC group followed by mixed and DC mode respectively (Table 2; Fig. 5).

**Table 2:** Number of failure modes in different obturation methods at different root levels

Obturation method	Root level	Failure modes		
		DC	PC	Mixed
CC	Apical	**5**	2	2
	Middle	**4**	2	3
	Coronal	1	**5**	3
NC	Apical	0	**7**	2
	Middle	0	**7**	2
	Coronal	0	**8**	1
AC	Apical	1	**8**	0
	Middle	1	**7**	1
	Coronal	1	**5**	3

*DC =* Dentine-Cement, *PC =* Post-Cement

## Discussion

The present study compared the fibre post retention strength and failure mode between the CC and AC groups. Post retention strength and failure mode were significantly different between the CC and AC groups. Therefore, the null hypothesis was not accepted.

The tooth selection criteria of the BL/MD width ratio and IAF size were set to minimise the various root canal shapes and sizes that could impact PBS testing [3, 19].

The common errors in the PBS results are from material deformity upon loading rather than the bond breaking at the material interfaces. This is caused by incorrect ratios of loading pin size/sample diameter and also sample thickness/diameter [25]. The present study strictly followed the ratios recommended by Chen et al. 2013. The stereomicroscope images also confirmed that the bonds did break at the material interfaces in any sample and no material deformity was documented. The PBS in the AC group was significantly higher than the CC group at the apical and coronal 1/3 levels. These results were in agreement with a previous study [8].

The hybrid layer and resin tags in the SEM analysis are a proxy for DTs patency. More hybrid layer and resin tags were observed in the AC and NC groups compared with the CC group. This means that there are more patent DTs in the AC and NC groups than the CC group. The hybrid layer & resin tags increase micromechanical retention [26]. This was corroborated by the failure mode analysis results (i.e., DC failure mode was found less in the AC and NC groups compared with the CC group) and the PBS analysis results (i.e., PBS was higher in the AC and NC groups than the CC) group.

PBS depends on the bond strength at two interfaces i.e., PC and DC. Because the bond strength is significantly affected by the types of post and cement [27],the current study minimised these effects by using the same post and resin cement in all groups. In the bond strength at the PC interface was lower than the lowest bond strength at the DC interface, it would not be possible to demonstrate different PBSs, which stems from different bond strengths at the DC interface (due to different obturation methods). This study, however, found that DC failures occured in the CC and AC groups, which indicates that the PC bond strength was not lower than the lowest DC bond strength.

The overall PBS in the AC group was significantly higher than that in the CC group and was not significantly different from that in the NC group, which is probably the maximum PBS that can be achieved.

The PBS at the middle 1/3 was significantly higher than the apical 1/3 in the CC group (Fig. 3), likely because there were more DTs with larger diameters in the middle 1/3 than the apical 1/3. This finding was consistent with previous studies [28, 29]. This may also be due to post better adaption (higher frictional retention) at this level than the coronal 1/3. Good adaption also increases bond strength by reducing the resin cement thickness and the likelihood of void formation. Moreover, thin resin cement reduces C-factors, minimises polymerization shrinkage and shrinkage stress.

The three-dimensional obturation of the root canal system is considered important for establishing an effective apical seal, which is believed to play a meaningful role in preventing apical microleakage and contributing to the success of endodontic treatment [30]. The absence of an apical seal may result in the remaining microorganisms leaking into the periradicular tissues, leading to apical periodontitis [31].

An apical seal is also crucial for the success of post-endodontic restorations. The absence of an apical seal may lead to tissue fluid percolating back into the root canal system in some cases, increasing water sorption within the root canal [32]. Water sorption in the region of the resin composite core build-up and quartz fibre posts may lead to a decline in bond strength. This might be associated with the water sorption of the resin monomer, potentially causing the bonds between the resin composite core build-up and quartz fibre posts to degrade. Additionally, it was observed that the epoxy resin matrix in the fibre post tends to swell after water sorption, and partial delamination of the quartz fibres from the epoxy resin matrix along the periphery of the fibre post can occur [33].

In this study, the obturation method employed utilized the continuous wave of condensation technique for the down-pack procedure to 4 mm from the working length [34]. The 4 mm length of the apical root canal filling in the AC and CC groups comprised a gutta-percha matched cone and root canal sealer. Consequently, it was anticipated that comparable obturation quality and apical seal would be achieved in the AC and CC groups.

The continuous wave of condensation technique employed in this study has been demonstrated by several studies to provide densely packed obturation material and improved sealability in the apical third of the root canal [35, 36]. Furthermore, when combined with a bioceramic sealer, this technique resulted in a success rate comparable to epoxy resin in endodontically treated teeth [37, 38]. The SEM results in the AC group in this study also demonstrated sealer penetration into the dentinal tubules in the apical third of the root canal. However, it is important to note that studies using a glucose leakage model and fluid filtration test revealed no significant correlation between an apical seal, a potential factor in preventing apical microleakage, and the extent of sealer penetration into the dentinal tubules [39, 40].

Achieving an apical seal in this study was expected through the use of densely packed obturation material from the continuous wave of condensation technique in the apical third of the root canal, which may suggest predictability for successful endodontic treatment and subsequent post-endodontic restorations.

### Strengths

The strength of the study was that the root canal shapes and sizes were controlled so that they would not impact PBS testing. Root canal obturation was done by a single operator using the operating microscope for precise BCS application. A total etch adhesive system and dual-cured resin cement were used to maximize post retention strength [41]. The failure mode analysis was done by evaluators who knew only the specimen number, but not the group to which it belonged. Moreover, the SEM analysis was done to demonstrate the interfaces in each group.

### Clinical relevance

Continuous wave downpack with a heated plugger to 3.5 − 4.5 mm from the WL provides the best adaptation of gutta-percha to the root canal wall [42]. The AC obturation method reduces the amount of BCS-occupied DTs at the apical 1/3 level of the post space. This may lead to high post retention strength. The AC obturation method may reduce the amount of BCS used and cost and has no impact on the apical seal [7]. This obturation method also reduces the amount of filling material used, time to backfill and minimizes the risk of perforation from post space preparation.

The SEM and PBS results and failure mode analysis demonstrated the importance of patent DTs to hybrid layer and resin tag formation, which led to high DC bond strength and post retention strength. The protocol used in the present study could be considered to maintain patent DTs because the AC obturation method because it may minimize the DTs occupied by BCS [8]. Additionally, it is recommended to use a total etch adhesive system to remove the smear layer [43].

### Limitations

Laboratory-based PBS testing may not entirely simulate the clinical situation. Resistance to post dislodgement is due to the cumulative bond strength on the entire root canal surfaces. The current study design could not take this into account because it was studied using 1 mm root thickness increments. A post may sustain the multi-directional forces in real clinical conditions, PBS testing, however, can only apply to a single-directional force parallel to the root long axis. In addition, the experiment set up could not take parafunctional habits into account.

### Future directions

Future studies may benefit from a finite element analysis (FEA) study. Because FEA is a computer model, variations in root shapes and sizes are not an issue, models can be identically generated. Other parameters can be varied to test their effects on the same model. Different root canal shapes, sizes, and coronal restoration types can be generated. Multi-directional, parafunctional forces can be studied. This allows the study to evaluate additional clinically relevant factors.

## Conclusions

The apical coating obturation method performed using an operating microscope had a significantly higher PBS than the conventional coating method. This may reduce the likelihood of fibre post dislodgement. This laboratory study, however, was only preliminary in nature. Therefore, clinical studies, are required to corroborate the results.

## Data Availability

The data that support the findings of this study are available from the corresponding author upon request.

## List of Abbreviations

BCS: Bioceramic sealer
DTs: Dentinal tubules
PUI: Passive ultrasonic irrigation
BL: Bucco-lingual
MD: Mesio-distal
NaOCl: Sodium hypochlorite
EDTA: Ethylene diamine tetra acetic acid
PUI: Passive ultrasonic irrigation
CC: Conventional coating
NC: Non coating
AC: Apical coating
SEM: Scanning electron microscope
PBS: Push out bond strength
DC: Dentine-cement
PC: Post-cement
MD: Mean difference
CI: Confidence interval
IAF: Initial apical file
MicroCT: Micro computed tomography
